# Diagnostic Utility of Neuregulin for Acute Coronary Syndrome

**DOI:** 10.1155/2016/8025271

**Published:** 2016-03-24

**Authors:** Maame Yaa A. B. Yiadom, Jeremy Greenberg, Holly M. Smith, Douglas B. Sawyer, Dandan Liu, Jahred Carlise, Laura Tortora, Alan B. Storrow

**Affiliations:** ^1^Department of Emergency Medicine, Vanderbilt University School of Medicine, 703 Oxford House, 1313 21st Avenue S., Nashville, TN 37232, USA; ^2^Department of Medicine, Division of Cardiovascular Medicine, Vanderbilt University School of Medicine, Nashville, TN 37203, USA; ^3^Department of Biostatistics, Vanderbilt University School of Medicine, Nashville, TN 37232, USA

## Abstract

The purpose of this study was to determine the diagnostic test characteristics of serum neuregulin-1*β* (NRG-1*β*) for the detection of acute coronary syndrome (ACS). We recruited emergency department patients presenting with signs and symptoms prompting an evaluation for ACS. Serum troponin and neuregulin-1*β* levels were compared between those who had a final discharge diagnosis of myocardial infarction (STEMI and NSTEMI) and those who did not, as well as those who more broadly had a final discharge diagnosis of ACS (STEMI, NSTEMI, and unstable angina). Of 319 study participants, 11% had evidence of myocardial infarction, and 19.7% had a final diagnosis of ACS. Patients with MI had median neuregulin levels of 0.16 ng/mL (IQR [0.16–24.54]). Compared to the median of those without MI, 1.46 ng/mL (IQR [0.16–15.02]), there was no significant difference in the distribution of results (*P* = 0.63). Median neuregulin levels for patients with ACS were 0.65 ng/mL (IQR [0.16–24.54]). There was no statistical significance compared to those without ACS who had a median of 1.40 ng/mL (IQR [0.16–14.19]) (*P* = 0.95). Neuregulin did not perform successfully as a biomarker for acute MI or ACS in the emergency department.

## 1. Introduction

Identifying a sensitive and specific screening test for the spectrum of acute coronary syndrome (ACS) is a significant ED challenge. High-sensitivity troponin assays have been evaluated as an aid in ED risk stratification. However, as these assays have been introduced into clinical practice, lower diagnostic thresholds are used. An increasing number of patients are identified with chronic cardiovascular comorbidities or acute nonischemic conditions [1–3], making the diagnosis of ACS more clinically challenging. Sensitivity for detection of MI is improved, but diagnostic specificity is decreased.

An adjunct or alternative biomarker to the cardiac troponins that increases the diagnostic specificity for myocardial infarction, while maintaining sensitivity, is desirable. In addition, a clinically useful biomarker for myocardial ischemia without evidence of infarction has not been reported. C-reactive protein (CRP) [4, 5], highly sensitive C-reactive protein (hsCRP), ischemia modified albumin [6, 7], and pro-*β* natriuretic peptide (proBNP) [8, 9] are markers that have showed promise. Yet there is still no definitive biomarker for unstable angina.

A new biomarker that is able to identify plaque rupture in a coronary artery that leads to cardiac ischemia, but prior to cardiac muscle injury, would provide clinicians with an invaluable tool in the diagnosis and management of ACS. Such a marker may offer promise for improved accuracy in diagnosis throughout the ACS spectrum.

Neuregulin-1*β* (NRG-1*β*) is a paracrine growth factor involved in cardiac embryogenesis and maintenance of the adult heart [10]. Preclinical studies demonstrate that myocardial ischemic injury activates NRG-1*β* [11]. Early clinical studies show a correlation between the NRG-1*β* levels and the severity of heart failure and coronary disease. In a heart failure cohort study of almost 900 patients, those with ischemic cardiomyopathy were found to have higher circulating NRG-1*β* compared to those with nonischemic cardiomyopathy [12]. An additional study of 60 patients undergoing cardiac catheterization demonstrated increased plasma NRG-1*β* levels in patients with stress-induced cardiac ischemia [13]. These studies have led to the hypothesis that NRG-1*β* may serve as a useful biomarker in the diagnosis of not only myocardial infarction, but myocardial ischemia, as represented in the spectrum of ACS.

The purpose of our pilot trial was to determine the diagnostic test characteristics of serum NRG-1*β* for the detection of ACS in ED patients with symptoms suggestive of acute ischemia.

## 2. Materials and Methods

### 2.1. Setting and Selection of Participant

We evaluated ED patients with signs and symptoms prompting an evaluation for ACS at a tertiary care hospital with >70,000 ED visits per year from November 2011 through February 2013. Patients were candidates if they had signs and symptoms prompting an ACS evaluation by the treating physician, a 12-lead ECG taken at time of presentation to the ED, and provided informed consent. Patients were excluded if they participated in the study previously or were actively engaged in an investigational device or drug study (with the exception of other diagnostic studies). This study was approved by the institutional review board.

### 2.2. Data Collection and Processing

Each patient's care was guided by the treating physician; the study sample was the first serum sample drawn during the ED visit and subsequently analyzed for troponin I (Siemens ADVIA Centaur® TnI-Ultra*™* assay). These serum samples are typically drawn within hours of arrival in the emergency department for acute care. Plasma samples were collected in EDTA vacutainer tubes at enrollment and stored at −80°C. At the time of analysis, the frozen samples were thawed in a single run. NRG-1*β* concentration in samples was measured using a DuoSet ELISA Development System from R&D Systems, Minneapolis, MN (cat# DY377), according to manufacturer's specifications at the time of this assay lot and as previously reported [11]. Subsets of samples were run at dilutions of 1 : 5 and 1 : 30. Concentrations were calculated from the 1 : 5 dilution unless the value was above the highest standard, in which case the 1 : 30 dilution was used. If values from the 1 : 30 dilution remained above the highest standard, they are reported as 314.5 ng/mL. If levels were undetectable, the values were set to the limit of detection defined as 1/2 the value of the lowest point on the standard curve.

### 2.3. Outcome Measures

The final outcome measure of this study was quantifying the correlation between the NRG-1*β* levels of patients with and without MI, as well as the broader diagnosis of ACS. The diagnosis of MI was based upon the final discharge diagnosis by the treating physician. This was the ED physician if the patient was discharged from the ED, and inpatient physician if the patient was admitted to an inpatient bed. Final diagnoses were abstracted from the electronic medical record by an investigator (Jeremy Greenberg) and adjudicated by the senior author (Alan B. Storrow).

### 2.4. Data Analysis

Descriptive statistics were used to identify the frequency of ACS and its component conditions (STEMI, NSTEMI, and UA) within the study population. We performed a logistic regression using software package R version 3.1.1 to evaluate whether NRG-1*β* predicts ACS or MI after controlling for known coronary artery disease risk factors of age and diabetes.

## 3. Results

### 3.1. Study Population Demographics

Of 319 study participants, 10.7% of the total population had evidence of myocardial infarction, and 19.7% had a final diagnosis of ACS. 50% of the group were male, 80% were Caucasian, and the median age was 56 years (IQR 48–65). Those with AMI (Table 1) had a median age of 62 years (IQR 55–78), compared to the non-MI group median of 55 years (IQR 47–64). Those with ACS (Table 2) had a median age of 59 years (IQR 54–74). There was no statistical difference among these groups (*P* < 0.001).

### 3.2. Neuregulin Performance

**Figure 1:**
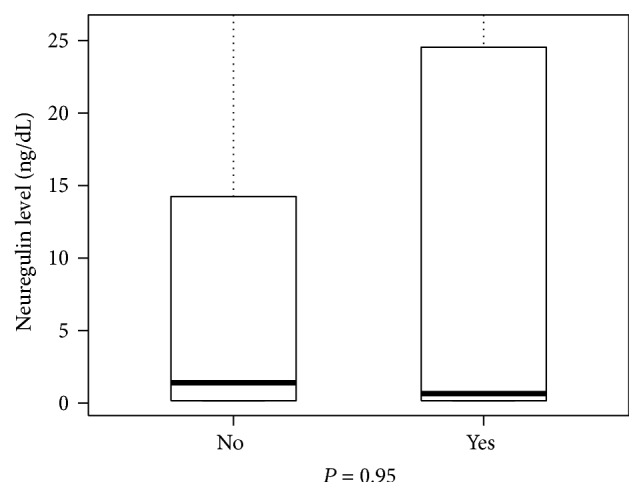
Box plot comparing neuregulin levels in patients with and without myocardial infarction.

**Figure 2:**
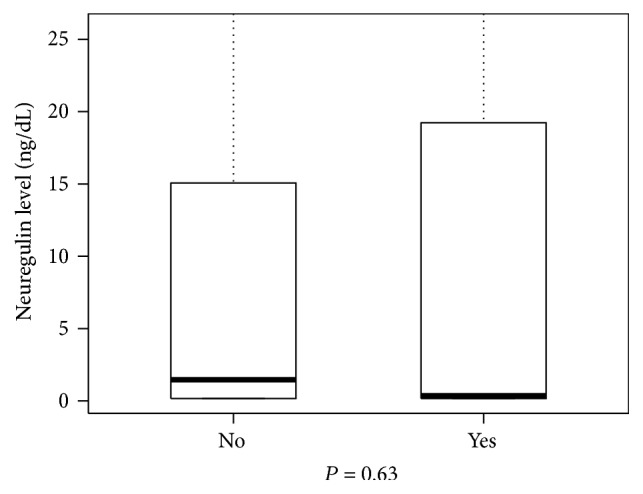
Box plot comparing neuregulin levels in patients with and without acute coronary syndrome.

Patients with a final diagnosis of MI had NRG-1*β* levels of 0.16 ng/mL (IQR [0.16–19.23]). Compared to those without MI 1.46 ng/mL (IQR [0.16–15.02]), there is a little distinction in the distribution of the neuregulin results (*P* = 0.63, Figure 1). The median NRG-1*β* levels for patients with a final diagnosis of ACS were 0.65 ng/mL (IQR [0.16–24.54]). Comparing this subpopulation to those without ACS 1.40 ng/mL (IQR [0.16–14.19]), we did not find a statistically significant difference (*P* = 0.95, Figure 2). We examined whether sex was a confounding factor and found that all 4 of these subgroups have the same median and IQR values, 0.94 ng/mL (0.16, 15.18).

**Figure 3:**
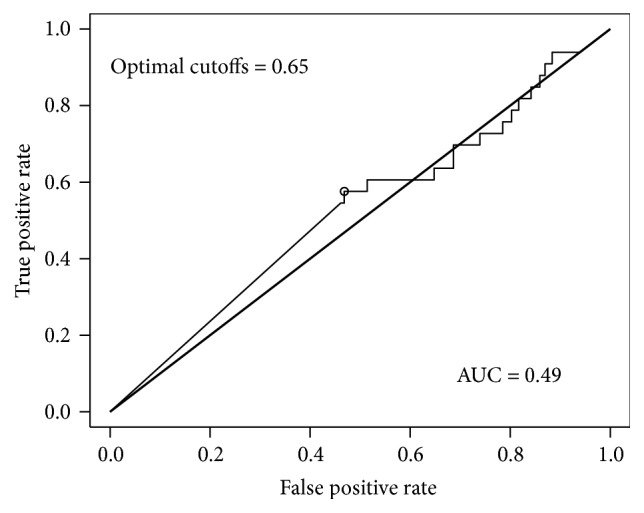
ROC curve for neuregulin in predicting MI.

**Figure 4:**
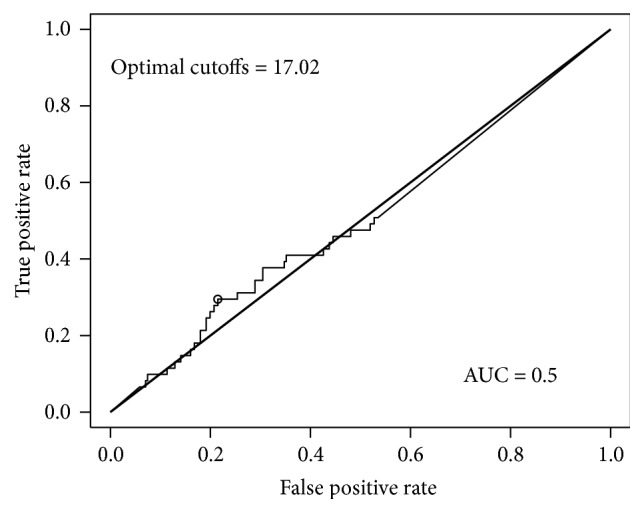
ROC curve for neuregulin in predicting ACS.

The receiver operator curve (ROC) AUC for NRG-1*β* in predicting MI and ACS was 0.49 (Figure 3) and 0.5 (Figure 4), respectively. Both values are near the line of no discrimination. This suggests that a single NRG-1*β* result at time of presentation is a poor biomarker for the diagnosis of MI or, more broadly, ACS.

### 3.3. Logistic Regression

Controlling for the influences of diabetes, age, and the diagnostic troponin level, we predicted MI in a patient with their NRG-1*β* level alone. This resulted in an odds ratio of 0.99 (*P* value = 0.027). Similarly, the odds ratio for predicting ACS was 1.01 (*P* = 0.67). This indicates that NRG-1*β* was nonpredictive for MI and ACS.

## 4. Discussion

There are more than 8 million visits a year to emergency departments (ED) for chest pain, or other symptoms concerning acute coronary syndrome [14]. Upon presentation to a healthcare provider, these patients are evaluated for one of the conditions along the spectrum of acute coronary syndrome (ACS).

Cardiac troponin (I or T) elevates in serum in the presence of myocardial injury. Values that exceed the 99th percentile threshold for the normal population are the gold-standard biomarker for the diagnosis of myocardial infarction [1]. The use of high-sensitivity cardiac troponin assays, which are commercially available but non-FDA approved for use in the United States, will identify more patients meeting the current definition of AMI at earlier time intervals [15–17]. Conversely, high-sensitivity assays capture more causes of myocardial injury and decrease specificity for myocardial infarction [18]. This raises concerns surrounding therapeutic choices of clinicians, who may incorrectly equate an abnormal troponin value to an AMI [19, 20].

Given these limitations, there arises a need for an additional biomarker to improve the early ED evaluation of patients with chest pain or other signs and symptoms suspicious for ACS. If such a marker was sufficiently sensitive and specific during the early development of ACS, it would provide substantial utility.

Despite past studies suggesting that circulating NRG-1*β* may increase with cardiac ischemia, we were unable to find any evidence to support its usefulness as a marker of ACS. One explanation for these negative findings involves the timing of NRG activation in ACS. In isolated perfused myocardium, NRG activation and release occur at some point after reperfusion [11]. Another potential explanation is that NRG may be increased in the setting of other illnesses, such as those involving lung or musculoskeletal injury. This could increase the incidence of NRG-1*β* elevation in the acute care population and decreased the specificity of ACS.

The proximity of this timing to an acute episode was our motivation for conducting the study in the emergency department setting. It is possible that neuregulin levels rise in a more delayed fashion. But this would make it a less valuable biomarker for the early diagnosis of acute coronary syndrome.

Our study was limited by the utilization of a single center, the lack of serial sampling, and the reliance of medical record diagnosis for our outcome measures. However, the overall extremely poor diagnostic performance provides evidence that larger and more methodologically robust studies would unlikely alter our results. Given NRG-1's physiologic attributes, it is possible that serial sampling, coupled with investigations into possible cardiac NRG-1 subtypes, might show some promise.

## 5. Conclusion

A single sample determination of NRG-1*β* at ED presentation is not predictive of a final diagnosis of MI or ACS.

## Figures and Tables

**Table 1:** Patients with and without myocardial infarction.

	Total patients		No MI		MI	
	319		285	**89.3%**	34	**10.7%**
Sex						
Male	167	52.4%	146	51.2%	21	61.8%
Female	152	47.6%	139	48.8%	13	38.2%
Age (years)						
All	56	IQR (48–65)	55	IQR (47–64)	56	IQR (55–78)
Race						
Caucasian	254	79.6%	50	17.5%	25	73.5%
African American	56	17.6%	1	0.4%	6	17.6%
Other	5	1.6%	1	0.4%	1	2.9%
Asian	1	0.3%	229	80.4%	0	0.0%
More than one	2	0.6%	4	1.4%	1	2.9%
Unidentified	1	0.3%	0	0.0%	1	2.9%

**Table 2:** Patients with and without acute coronary syndrome.

	Total patients		No ACS		ACS	
	319		256	**80.3%**	63	**19.7%**
Sex						
Male	167	52.4%	129	50.4%	38	60.3%
Female	152	47.6%	127	49.6%	25	39.7%
Age (years)						
All	56	IQR (48–65)	54	IQR (46–63)	59	IQR (54–74)
Race						
Caucasian	254	79.6%	202	78.9%	52	82.5%
African American	56	17.6%	48	18.8%	8	12.7%
Other	5	1.6%	4	1.6%	1	1.6%
Asian	1	0.3%	1	0.4%	0	0.0%
More than one	2	0.6%	1	0.4%	1	1.6%
Unidentified	1	0.3%	0	0.0%	1	1.6%

## Abbreviations

ED: Emergency department
MI: Myocardial infarction
ACS: Acute coronary syndrome
STEMI: ST segment elevation myocardial infarction
NSTEMI: Non-ST segment elevation myocardial infarction
UA: Unstable angina
NRG-1*β*: Neuregulin-1*β*
VEGF: Vascular endothelial growth factor.
